# The oral microbiome in young women at different stages of periodontitis: *Prevotella* dominant in stage III periodontitis

**DOI:** 10.3389/fcimb.2022.1047607

**Published:** 2022-12-01

**Authors:** Yaqiong Zhao, Yunzhi Feng, Qin Ye, Jing Hu, Yao Feng, Zeyue Ouyang, Jie Zhao, Yun Chen, Li Tan, Ningxin Chen, Marie Aimee Dusenge, Xiaolin Su, Yue Guo

**Affiliations:** Department of Stomatology, The Second Xiangya Hospital, Central South University, Changsha, Hunan, China

**Keywords:** periodontitis, 16S rRNA, *Prevotella intermedia*, LPS, endoplasmic reticulum

## Abstract

**Objective:**

Periodontitis progression is related to the dynamic dysbiosis of oral microbiome. We identified the dominant bacteria and the potential pathway in young women with stage-III periodontitis.

**Materials and methods:**

Samples of subgingival plaque were collected from 26 young women with periodontitis (20 with stage-I and 6 with stage-III). Using 16S rRNA-sequencing, we determined the variation in oral bacterial communities of the two groups, and identified the dominant bacteria of each group. We used the Kyoto Encyclopedia of Genes and Genomes (KEGG) database to evaluate the signaling pathways related to the difference in oral bacterial composition. The role of the dominant bacteria of stage-III periodontitis was investigated *in vivo* and *in vitro* using an endoplasmic reticulum stress inhibitor.

**Results:**

Young women with stage-I periodontitis had higher values for the Chao1 Index, Observed Species and Phylogenetic Diversity Whole Tree Index than those for women with stage-III periodontitis. β-diversity analyses revealed that samples could be divided into different groups according to the periodontitis stage. The most representative biomarkers of stage-III periodontitis in young women were bacteria of the phylum Bacteroidetes, its order, family and genera *Bacteroidales*, *Prevotellaceae* and *Prevotella*. The KEGG database revealed that the change in oral bacterial composition of young women with stage-III periodontitis may be related to protein processing in an endoplasmic reticulum pathway. Salubrinal (an endoplasmic reticulum stress regulator) controlled expression of Runx2, Col1a1, Ocn in mouse bone-marrow mesenchymal cells. Salubrinal administration showed that moderate endoplasmic reticulum stress inhibited alveolar bone loss in periodontitis induced by *Prevotella intermedia* lipopolysaccharide.

**Conclusion:**

Differences between periodontitis stages were noted and bacteria of *Prevotella* species were abundant in young women with stage-III periodontitis. This phenomenon was related to protein processing in an endoplasmic reticulum pathway.

## Introduction

Many types of microorganisms reside in the human oral cavity. They include bacteria, fungi, viruses and archaea, which make up a rich microbial community known as the “oral microbiome” (Zorba et al., 2020). More than 1000 species of bacteria have been found to colonize different surfaces of the gingiva, teeth and tongue (Human Microbiome Project, 2012). In health, the oral microbiome and the host are in a state of dynamic homeostatic equilibrium. However, under certain conditions, such as reduced immunity and fluctuations in hormone levels, this equilibrium is disturbed, which can lead to a series of diseases, such as gingivitis, periodontitis or systemic diseases, such as diabetes, cardiovascular diseases, and adverse pregnancy outcomes (Kumar et al., 2003; Sampaio-Maia et al., 2016; Dioguardi et al., 2020).

Sex steroid hormones (SHHs) are cholesterol-derived molecules secreted into saliva and which reach oral tissues such as the gums and periodontium (Cornejo Ulloa et al., 2021). The prevalence and severity of periodontitis is lower in females in most age groups compared with those in males, which may be related to different SHH levels in males and females (Albandar, 2002). SHHs can regulate the composition and behavior of oral microorganisms, which is an important factor in oral microbiomes dysbiosis. Previously, we revealed sex-based variations in the oral microbiomes with regard to periodontitis (Zhao et al., 2021b; Zhao et al., 2021a). The dominant bacteria in males with severe periodontitis were those of the genera *Pseudomonas* and *Papillibacter* whereas, in females, they were from the genus *Tannerella* and order Fusobacteriales (Zhao et al., 2021b). For older patients with periodontitis, the predominant bacterial genus was *Haemophilus* in males and *Campylobacter* in females (Zhao et al., 2021a).

Endoplasmic reticulum (ER) stress refers to disequilibrium between the cellular demand for the function and capacity of the ER. ER stress participates in inflammation, bone loss and degradation of the extracellular matrix. Upregulation of the ER stress response in periodontitis has been observed (Domon et al., 2009). ER stress induced by *Porphyromonas gingivalis* has been shown to cause alveolar bone loss in an experimental model of periodontitis (Yamada et al., 2015). Kimura and colleagues found that salubrinal (SAL) (2.0 mg/kg, once-daily) alleviated ER stress and inhibited alveolar bone loss in an experimental model of periodontitis (Kimura et al., 2021). SAL is a synthetic compound that can reduce ER stress by increasing phosphorylation of eIF2a by suppressing protein phosphatase (Boyce et al., 2005). SAL can upregulate expression of Runx2, Ocn and Col1α1 to induce osteoblast differentiation (Tanaka et al., 2014). Previously, we found that ER stress occurred after tooth extraction, and that a moderate degree of ER stress promoted healing of alveolar bone after SAL treatment. However, the regulatory effect of different concentrations of SAL on periodontitis has not been studied. It has been reported that ER stress is also affected by hormones and age, but the mechanism of action is not known (Hu et al., 2016; Yang et al., 2018; Gil-Hernández and Silva-Palacios, 2020).

In the current study, samples of subgingival plaque from young women with stage-I or stage-III periodontitis were obtained. 16S rRNA-sequencing revealed the dominant bacteria of stage-III periodontitis were those of the genus *Prevotella*. Analyses of enrichment of signaling pathways using the Kyoto Encyclopedia of Genes and Genomes (KEGG) database revealed the dominant pathway of stage-III periodontitis to be “protein processing in endoplasmic reticulum”. The regulatory effect of ER stress on periodontitis caused by lipopolysaccharide (LPS) from *Prevotella intermedia* was demonstrated *in vivo* and *vitro*.

## Materials and methods

### Ethical approval of the study protocol

The study protocol was approved by the Ethics Committee of The Second Xiangya Hospital of Central South University (Changsha, China). Recruited patients provided written informed consent. The clinical trial registration number is ChiCTR2100046828.

### Exclusion criteria

Exclusion criteria were: (i) periodontal treatment in the last 6 months; (ii) tobacco smoking; (iii) systemic disease; (iv) use of antibiotics within the previous 1 month; (v) trauma; (vi) recent surgery; (vi) pregnancy.

### Participants

Study participants were women aged 20–44 years with ≥15 teeth. The stages of periodontal disease were determined based on the standard of the Staging and Grading Periodontitis of The American Academy of Periodontology (AAE) (Tonetti et al., 2018). The clinical attachment level at the site of greatest tooth loss was recorded: 1–2 mm was defined as stage I, 3–4 mm as stage II and ≥5 mm as stage III. Loss ≤4 teeth was recorded as stage III whereas loss ≥5 teeth was diagnosed as stage IV. Patients with stage-III or stage-IV periodontitis were combined into a stage-III group.

### Collection of subgingival plaque and extraction of bacterial DNA

Samples of subgingival plaque of the four first molars were collected using a sterile Gracey scraper. Next, they were added to phosphate-buffered saline (PBS), frozen immediately and stored at −80°C. DNA from samples was extracted and quantified using the DNeasy PowerSoil Kit (Qiagen, Hilden, Germany) and a spectrometer (NanoDrop™ 2000; Thermo Fisher Scientific, Waltham, MA, USA). DNA quality was determined by agarose-gel electrophoresis.

### Sequencing of the V3-V4 region of 16S rRNA

16S rRNA-sequencing was done by OE Biotech (Shanghai, China). The polymerase chain reaction (PCR) primers 343 forward (5′-TACGGRAGGCAGCAG-3′) and 798 reverse (5′-AGGGTATCTAATCCT-3′) were used to amplify the V3–V4 region. Thermal cycling conditions were an initial denaturation at 94°C for 5 min followed by 26 cycles of 94°C for 30 s (denaturation), 55°C for 30s (annealing) and 72°C for 20 s (elongation), with a final extension at 72°C for 5 min. Amplified products were detected by electrophoresis using 1% agarose gels purified with AMPure XP beads (Agencourt; Beckman Coulter, Fullerton, CA, USA), and quantified using the Qubit dsDNA Assay Kit (Life Technologies, Carlsbad, CA, USA). Amplified samples were pooled for sequencing on the MiSeq platform (Illumina, San Diego, CA, USA). Raw paired-end reads were analyzed by QIIME 1.9.1. Sequences were clustered into identical operational taxonomic units (OTUs) using VSEARCH 2.4.2 with ≥ 97% identity. OTU taxonomy was analyzed using a naive Bayesian classifier (Ribosomal Database Project classifier) and the SILVA database (www.arb-silva.de/).

### Bioinformatics analysis

α-diversity was measured using various indices (observed species, Chao, Shannon, Simpson, Good’s Coverage, Phylogenetic Diversity (PD) Whole Tree) and was compared by the Student’s t-test and Kruskal–Wallis test. β-diversity was analyzed by principal coordinates analysis (PCoA) and nonmetric multidimensional scaling (NMDS) based on unweighted UniFrac distances. The relative abundance of dominant bacteria between different groups was compared using the Wilcoxon rank-sum test. Linear discriminant analysis effect size (LEfSe) was undertaken to discover different biomarkers. The KEGG database (www.genome.jp/kegg/) was used to predict the potential function of subgingival microbiota.

### Preparation of LPS and SAL

LPS from lyophilized *P. intermedia* was purified by the standard hot phenol–water method followed by treatment with proteinase K, DNase and RNase to eliminate contaminating proteins and nucleic acids, as described by Choi and colleagues (Choi et al., 2014). The experimental steps were completed by the Shanghai Bioresource Collection Center (Shanghai, China). SAL (Tsbiochem, Shanghai, China) was dissolved in dimethyl sulfoxide.

### Cell culture and LPS treatment

Mouse bone-marrow mesenchymal cells (mBMSCs) were cultivated in minimum essential medium-alpha supplemented with 10% fetal bovine serum and penicillin (100 U/mL) and streptomycin (100 μg/mL) at 37°C in a humidified incubator in an atmosphere of 5% CO_2_. Cells were seeded in six-well culture plates with 2 × 10^6^ cells/well and incubated for 24 h to adhere. Then, the cells were treated with *P. intermedia* LPS (20 μL/well) with/without SAL (20, 100, 200 μM) for an additional 24 h.

### Real-time reverse transcription-quantitative polymerase chain reaction (RT-PCR)

After incubation, total RNA was isolated using TRIzol^®^ Reagent (Invitrogen, Carlsbad, CA, USA) according to manufacturer instructions. Synthesis of complementary-DNA from total RNA was conducted using the PrimeScript™ Strand cDNA Synthesis Kit (Takara Biotechnology, Shiga, Japan). RT-PCR was carried out using a TB Green^®^ Premix Ex Taq™ II kit (Takara Biotechnology) with the LightCycler^®^ 96 system (Roche, Basel, Switzerland). With regard to thermal cycling, after denaturing at 95°C for 30 s, PCR was undertaken for 40 cycles of 95°C for 5 s and 60°C for 20 s. Expression of β-actin served as the internal control. The sequences of primers (forward and reverse, respectively) were 5′-CCTTCAAGGTTGTAGCCCTC-3′ and 5′-GGAGTAGTTCTCATCATTCCCG-3′ for Runx2; 5′-TGAACGTGGTGTACAAGGTC-3′ and 5′- CCATCTTTACCAGGAGAACCAT-3′ for Col1a1; 5′- CAAGCAGGAGGGCAATAAGGTAGTG-3′ and 5′-CGGTCTTCAAGCCATACTGGTCTG-3′ for Bglap.

### Immunoblotting

Total protein from cells was prepared using lysis buffer, followed by heating to 100°C for 5 min and then storage at −80°C. Protein samples (20 µg) for each lane underwent sodium dodecyl sulfate–polyacrylamide gel electrophoresis on 12% gels and were transferred onto nitrocellulose membranes. The latter were probed with primary antibodies (all at 1:1000 dilution) at 4°C overnight: COL1A1 (Affinity Biosciences, Cincinnati, OH, USA), RUNX2 (Abcam, Cambridge, UK), OCN (Abcam) and β-actin (Proteintech, Chicago, IL, USA). Then, nitrocellulose membranes were incubated with the corresponding peroxidase-conjugated secondary antibodies. Immunoreactive protein bands were detected using NcmECL Ultra Reagent (NCM Biotech, Suzhou, China).

### Animals

Eight-week-old C57BL/6J mice (SJA Laboratory Animals, Changsha, China) were housed under specific pathogen-free conditions with food and tap water available ad libitum. The protocol for animal experiments was approved by the Experimental Animal Ethics Committee of Second Xiangya Hospital.

### Gingival injection of LPS and SAL administration

Mice were divided into four groups. One group received 5 μg of a solution of *P. intermedia* LPS (1 mg/mL) injected into the gingiva *via* a syringe (1700 series; Hamilton, Bonadoz, Switzerland) around the mandibular first molar every 48 h for 13 times in total. One group received a gingival injection of *P. intermedia* LPS plus an intraperitoneal injection of SAL (0.143 mg/kg) 2 h before LPS injection. One group received a gingival injection of *P. intermedia* LPS plus an intraperitoneal injection of DMSO 2 h before *P. intermedia* LPS injection. The control group was given a gingival injection of PBS only. Before injection of LPS, anesthesia was induced with 1% pentobarbital sodium.

### Histology

Twenty-four hours after the 13th injection, the bilateral mandibles were removed surgically, dissected and fixed in 4% paraformaldehyde phosphate buffer solution for 48 h. Samples were decalcified with 17% EDTA for 21 days and then embedded in paraffin. Sections of thickness 4 μm were prepared in a mesiodistal orientation. Sections presenting the entire molar roots were selected for staining (hematoxylin and eosin (H&E)) and imaged on a Scan Scope GL optical microscope (Axiocam 503 color; Zeiss, Wetzlar, Germany).

### Staining with alkaline phosphatase (ALP)

After deparaffinization and rehydration, sections of thickness 4 μm were treated with an incubation solution (sodium pentobarbital (0.2 g), calcium chloride (0.2 g), β-sodium glycerophosphate (0.3 g), magnesium sulfate (0.4 g) dissolved in double-distilled water (10 mL)) for 3 h at 37°C. Then, sections were incubated with 2% cobalt nitrate for 5 min, treated with ammonium sulfate solution for 10 s and counterstained with 2% safranin. After dehydration with a graded series of alcohol solutions, sections were observed under light microscopy. Dark-brown particles were identified as positive-stained areas.

### Statistical analysis

Data are the mean ± standard deviation. Statistical analyses were carried out using one-way analysis of variance. A comparison of two groups was assessed by Tukey’s *post hoc* method. P < 0.05 was considered significant.

## Results

### General information of participants

Patients with stage-I periodontitis had a mean age of 33.10 (range, 27.38−38.82) years. Patients with stage-III periodontitis had a mean age of 34.17 (range 26.68−41.66) years. There was no significant difference in age between stage-I and -III groups (P > 0.05).

### Composition of the oral microbiota varied according to the periodontitis stage

**Figure 1:**
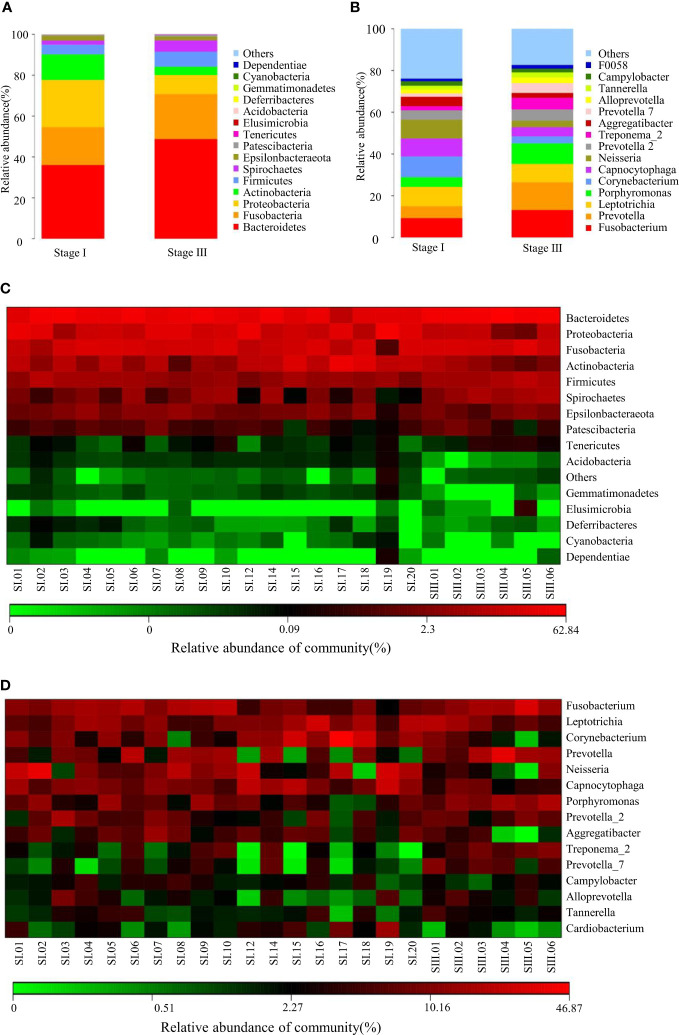
Composition of the oral microbiome between samples from women with stage-I or stage-III periodontitis at phylum **(A)** and genus **(B)**levels. Heatmaps at phylum **(C)** and genus **(D)** levels: the horizontal denotes sample information, and the vertical denotes information on species annotation. Colors indicate the relative abundance of a microbial community.

The relative abundance of oral microbiota was assessed at the level of the top-15 phyla and genera (**Figures 1A, B**). Compared with young women with stage-I periodontitis, the relative abundance of bacteria of the phylum *Bacteroidetes*, *Fusobacteria*, *Firmicutes* and *Spirochaetes* increased, whereas those of *Proteobacteria* and *Actinobacteria* decreased, in young women with stage-III periodontitis. At the genus level, the relative abundance of bacteria from *Fusobacterium*, *Prevotella*, *Porphyromonas*, *Prevotella 2*, *Treponema_2*, *Prevotella 7* and *Alloprevotella* was higher in the stage-III group than that in the stage-I group, whereas the relative abundance of bacteria from *Leptotrichia*, *Corynebacterium*, *Capnocytophaga*, *Neisseria* and *Aggregatibacter* in the stage-III group was lower than that in the stage-I group. The difference between patients with stage-I and -III periodontitis with regard to the relative abundance at phylum and genus levels is shown as a heatmap in **Figures 1C, D**.

### Young women with stage-III periodontitis had a lower richness of oral bacteria compared with that in women with stage-I periodontitis

**Figure 2:**
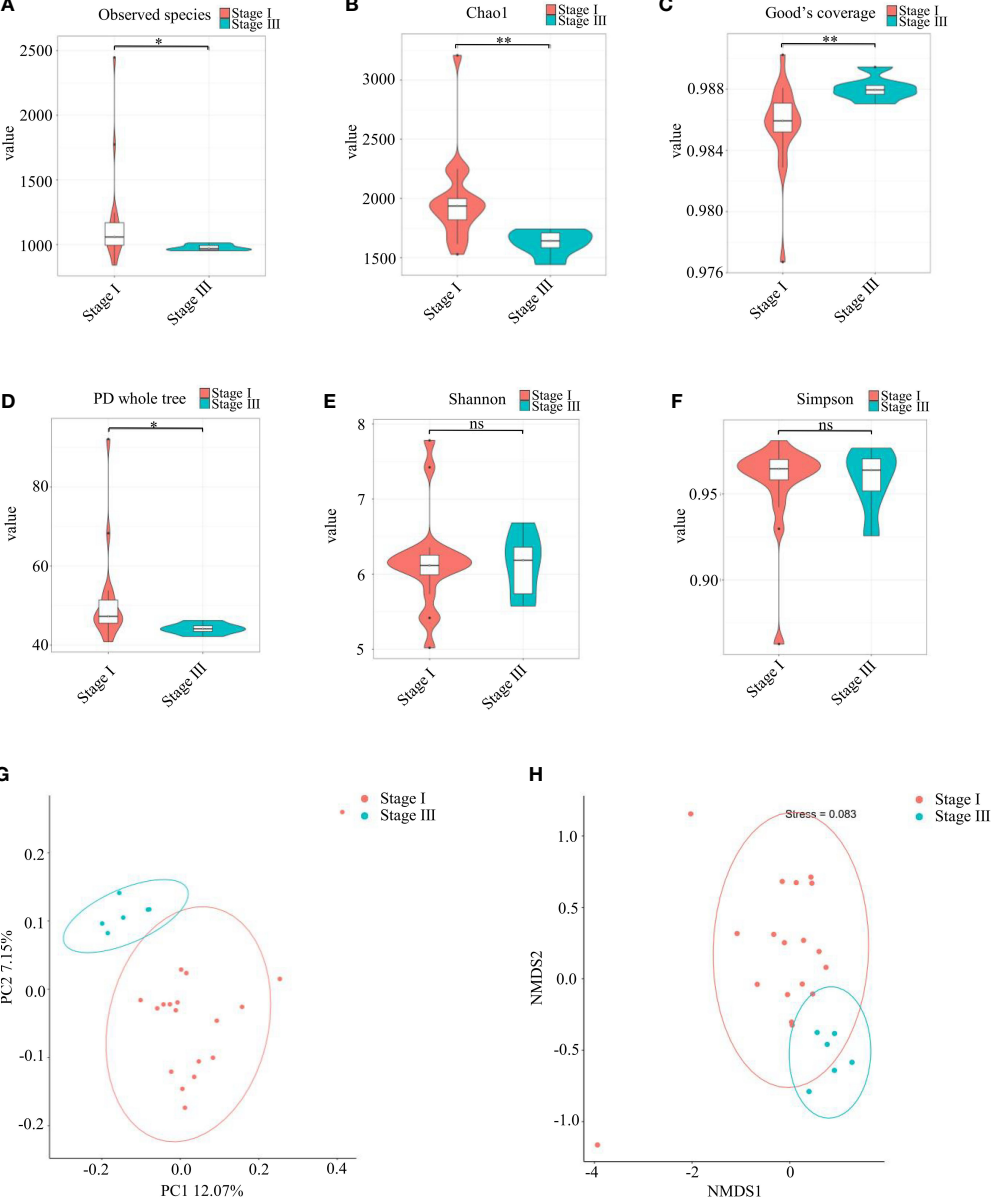
Analyses of α-diversity and β-diversity. **(A–F)** Violin plots of α-diversity indices (Observed species, Chao1, Good’s Coverage, PD Whole Tree, Simpson, Shannon) comparing stage I (red) and stage III (green) samples. (∗∗∗P <0.001, ∗∗P <0.01) ns, no significance. Based on the unweighted UniFrac distance, **(G)** principal coordinates analysis (PCoA) and **(H)** non-metric multidimensional scaling (NMDS) were used to analyze similarity in microbial communities.

The observed species and Chao1 Index were used to evaluate community richness, which were higher in the stage-I group compared with that in the stage-III group (P < 0.05) (**Figures 2A, B**). The Good’s Coverage Index reached 0.986 and 0.988 in the stage-I group and stage-III group, respectively, which indicated a higher probability of species in the stage-III group (P < 0.05, **Figure 2C**). The PD Whole Tree Index is an index to measure diversity that takes into account evolutionary distance: it was higher in the stage-I group (P < 0.05) (**Figure 2D**). No significant difference was observed in the Shannon Index or Simpson Index in terms of diversity (P > 0.05) (**Figures 2E, F**).

Assessment of variation in composition of oral microbial colonies in young women with stage-I or stage-III periodontitis was done based on β-diversity (**Figures 2G, H**). PCoA (**Figure 2G**) and NMDS (**Figure 2H**) revealed that the composition of the oral bacterial community in young women varied according to the stage of periodontitis.

### *Prevotella* was the dominant genus in young women with stage-III periodontitis

**Figure 3:**
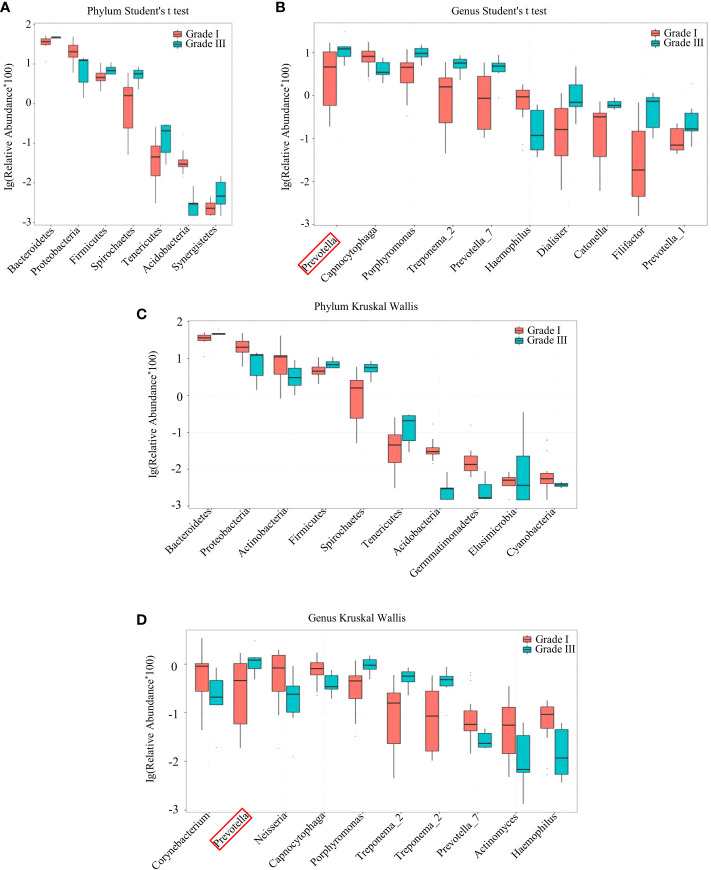
Species difference between samples from women with stage-I and stage-III periodontitis by the Student’s t-test **(A, B)** and Kruskal-Wallis test **(C, D)** at the phylum **(A, C)** and genus **(B, D)** levels.

Analyses of species differences between young women with stage-I or stage-III periodontitis were done by the Student’s t-test (**Figures 3A, B**) and Kruskal–Wallis test (**Figures 3C, D**). Those tests revealed the relative abundance of bacteria of the phylum *Bacteroidetes* and genus *Prevotella* to be higher in young women with stage-III periodontitis, whereas the relative abundance of bacteria of the phylum Proteobacteria was higher in young women with stage-I periodontitis.

**Figure 4:**
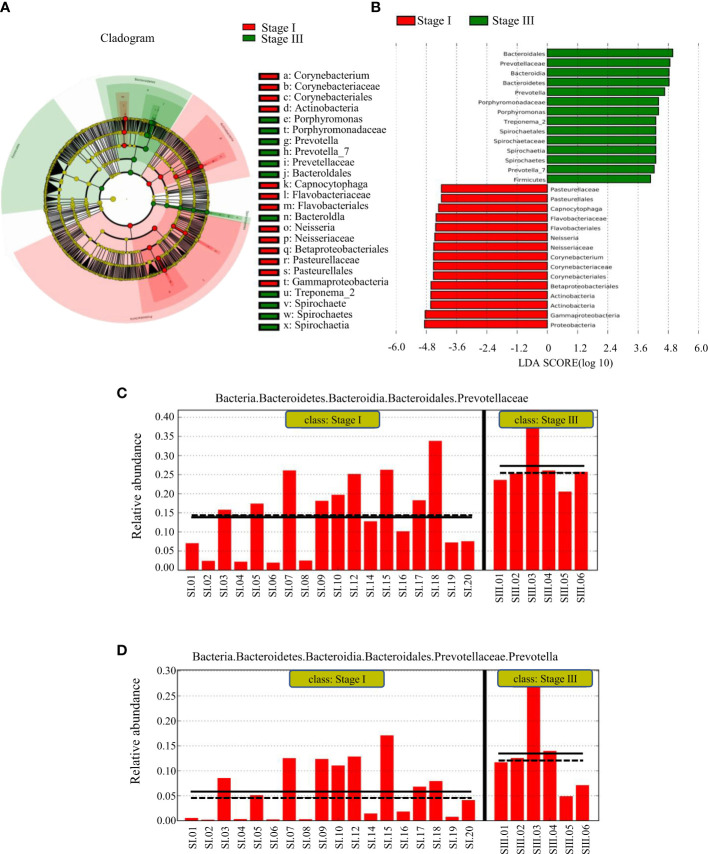
LEfSe results on the oral microbiome in different groups. **(A)** Histogram of the LDA scores between women with stage-I or stage-III periodontitis. Samples from women with stage-I periodontitis enriched taxa are indicated with a positive LDA score (green), and taxa enriched in samples from women with stage-III periodontitis have a negative score (red). The threshold on the logarithmic LDA score for discriminative features was set to 3.0. **(B)** Taxonomic cladogram obtained from LEfSe. The cladogram reports the taxa (highlighted by small circles and by shading; red indicating samples of stage-I periodontitis, green indicating samples of stage-III periodontitis and yellow indicating a non-significant relationship) showing different abundance values. **(C, D)** represent predominant biomarkers in patients with stage-III periodontitis.

LEfSe was used to identify the taxa most likely to be different in the oral bacterial community between young women with stage-I or -III periodontitis. The variation of enriched OM was identified at phylum and genus levels (**Figures 4A, B**). The predominant biomarkers in patients with stage-III periodontitis were analyzed (**Figures 4C, D**). Bacteria of the phylum Bacteroidetes, its order, family and genera *Bacteroidales*, *Prevotellaceae* and *Prevotella* were abundant in young women with stage-III periodontitis. Bacteria of the phylum *Proteobacteria* and its class *Gammaproteobacteria*, along with the phylum *Actinobacteria* and its class *Actinobacteria*, were abundant in young women with stage-I periodontitis. Bacteria of the genus *Prevotella* could be biomarkers for young women with stage-III periodontitis.

### Protein processing in an ER pathway was increased in women with stage-III periodontitis

**Figure 5:**
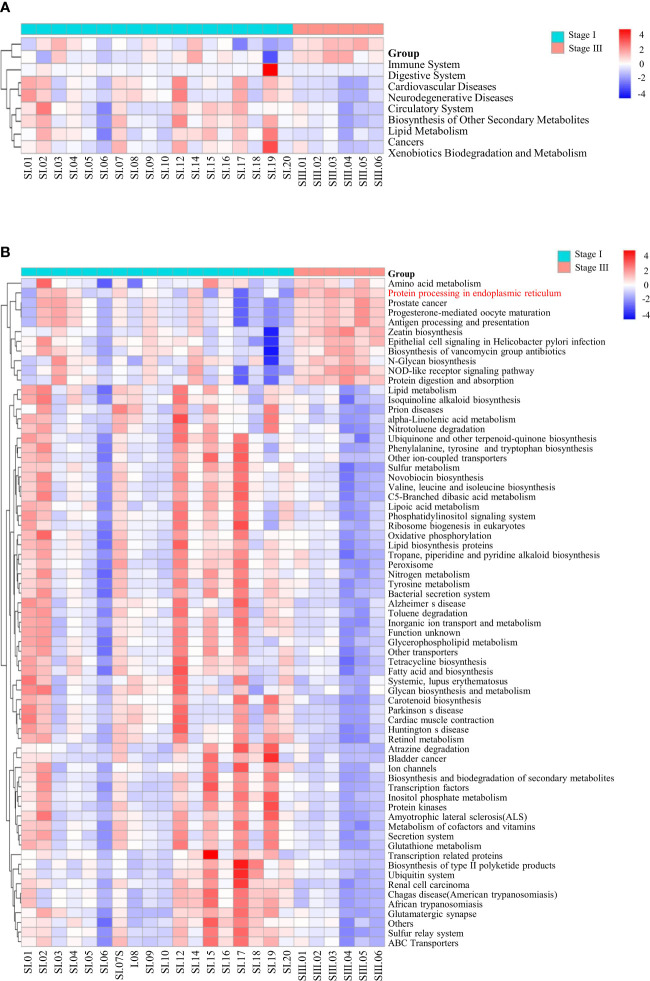
Pathway enrichment analysis. At the L2 **(A)** and L3 **(B)** level, using the KEGG database, the differences in all samples of women with stage-I or stage-III periodontitis were clustered into heatmaps.

The function of oral microbial communities was analyzed using QIIME2 and PICRUSt2. At the L2 level, the signaling pathways associated with the immune system and digestive system were enriched in patients with stage-III periodontitis (**Figure 5A**). At the L3 level, using the KEGG database, functions associated with amino-acid metabolism and protein processing in the ER were increased in women with stage-III periodontitis (**Figure 5B**).

### ER stress led to increased expression of RUNX2, OCN and COL1A1 in mBMSCs treated with *P. intermedia* LPS

**Figure 6:**
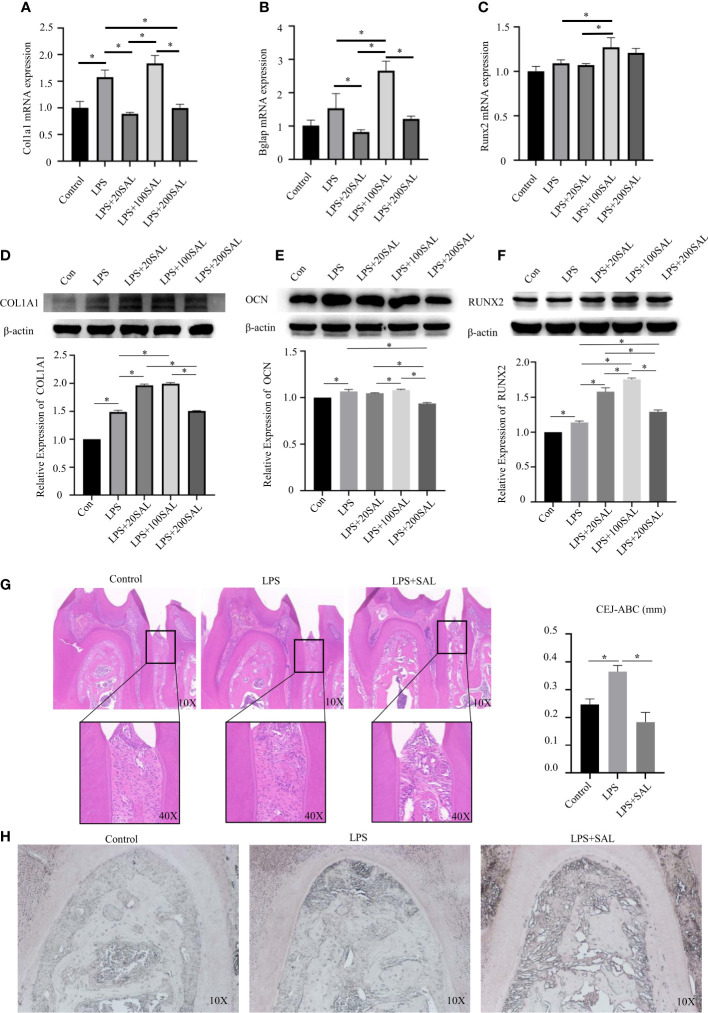
Effect of SAL on *P. intermedia* LPS-induced periodontitis *in vivo* and *in vitro*. **(A-C)** The mRNA expression of Runx2, Bglap, and Col1a1, respectively, in mBMSCs treated with *P. intermedia* LPS and different concentrations of SAL. **(D-F)** Representative western blots images and semi-quantitative analyses of the protein expression of COL1A1, OCN and RUNX2 in mBMSCs. **(G)** Representative H&E images of the periodontium after treatment with different drugs. **(H)** Representative images of ALP staining in the root bifurcation region *p < 0.05.

After LPS stimulation, mRNA expression of Col1a1 (**Figure 6A**) was increased significantly (P < 0.05), whereas expression of Bglap (**Figure 6B**) and Runx2 (**Figure 6C**) was increased slightly, but not significantly so. Protein expression of COL1A1, OCN and RUNX2 was upregulated after LPS stimulation (P < 0.05) (**Figures 6D–F**).

Then, cells were treated with different doses of SAL in the presence of LPS. SAL (20 and 200 μM) groups had similar mRNA expression of Col1a1 (P > 0.05) (**Figure 6A**). The 100-μM SAL group had similar mRNA expression of Col1a1 as that of the LPS group (P > 0.05), which was higher than that of the 20- and 200-μM SAL groups (P < 0.05) (**Figure 6A**). Protein expression of COL1A1 was higher in the 20- and 100-μM SAL groups than that in the LPS group and 200-μM SAL group (P < 0.05) (**Figure 6D**).

The highest mRNA expression of Bglap appeared in the 100-μM SAL group compared with that in the other groups (P < 0.0.5) (**Figure 6B**). The 20- and 200-μM SAL groups had similar mRNA expression of Bglap (P > 0.05), which was lower than that in the LPS group (P < 0.05) (**Figure 6B**). The 100-μM SAL group had higher protein expression of OCN compared with that in the 20- and 200-μM SAL groups (P < 0.0.5) (**Figure 6E**).

The 100-μM SAL group had higher mRNA expression of Runx2 compared with that in the LPS group or 20-μM SAL group (P < 0.05) (**Figure 6C**). RUNX2 expression from high to low was in the order 100, 20 and 200 μM of SAL, all of which were higher than that in the LPS group (P < 0.05) (**Figure 6F**).

### SAL inhibited alveolar bone loss in periodontitis induced by *P. intermedia* LPS by regulating ER stress

**Figure 7:**
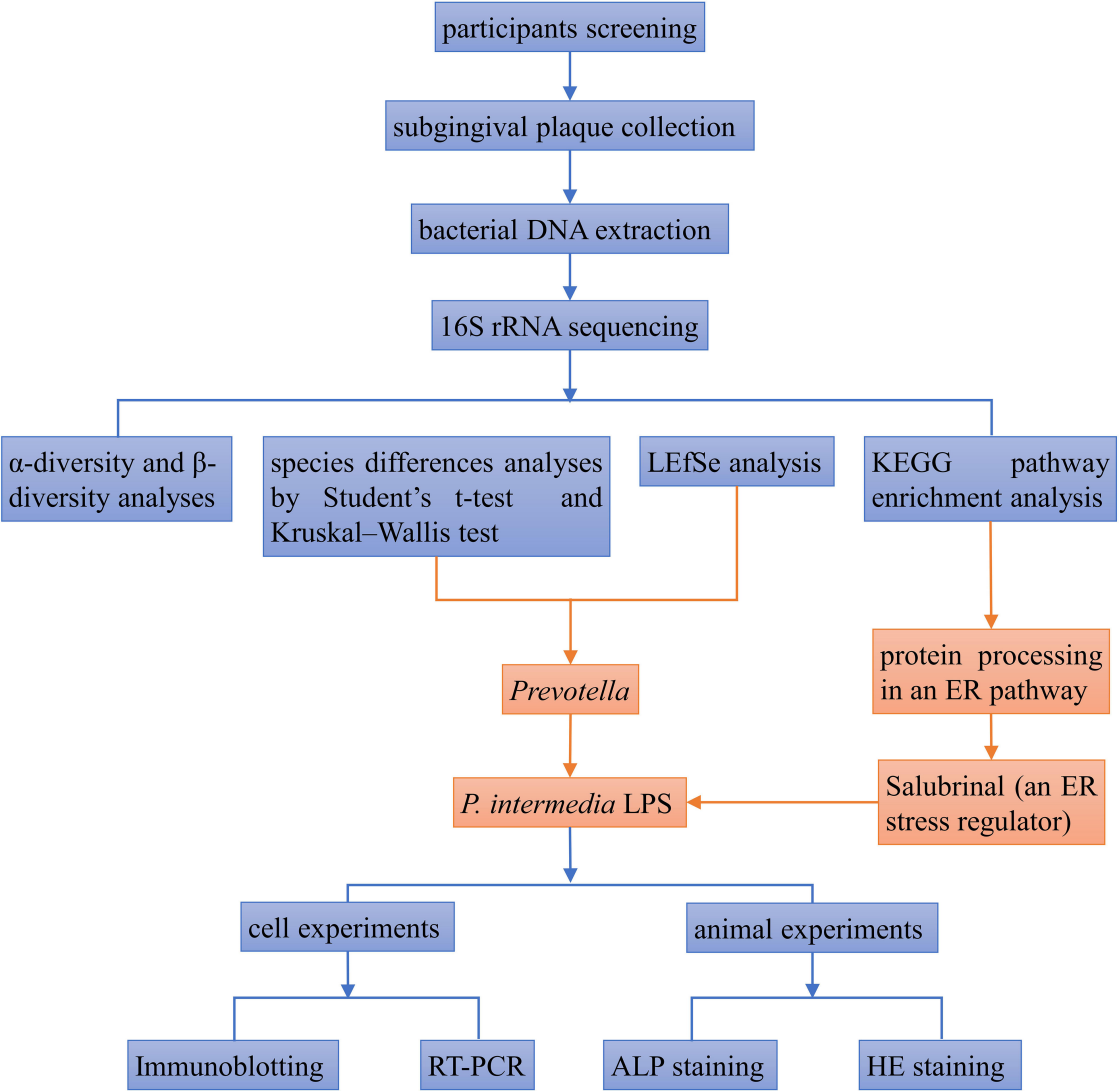
The flowchart of the experimental procedure.

The schedule used to induce periodontitis and SAL treatment is shown as **Figure 7**. Histology of periodontal-tissue specimens revealed differences in periodontal structure and bone mass between the first molar and second molar according to H&E staining (**Figure 6G**). Images (×40 magnification) showed that the distance from the cement–enamel junction to the alveolar bone crest in the LPS group was higher than that in the control group and SAL group (**Figure 6G**). ALP staining showed that, compared with the control group, ALP expression increased in the LPS group and SAL group, and was especially high in the SAL group (**Figure 6H**).

## Discussion

We discovered differences in the composition and abundance of oral bacteria of young women with stage-I or -III periodontitis. Women with stage-I periodontitis had higher α-diversity. Analyses of β-diversity indicated that samples could be divided into different groups according to the periodontitis stage. Bacteria of the genus *Prevotella* could be biomarkers in young women with stage-III periodontitis based on the Student’s t-test, Kruskal–Wallis test and LEfSe. The KEGG database suggested that the change in the oral bacterial community in young women with stage-III periodontitis may be related to protein processing in an ER pathway. Next, we applied SAL a regulator of ER stress, to explore its effect on periodontitis caused by *P. intermedia* LPS. Administration showed that moderate ER stress could inhibit alveolar bone loss in periodontitis by upregulating expression of RUNX2, COL1A1 and OCN.

Studies have shown that *Streptococcus gordonii* was more dominant in oral bioflim of males; *Porphyromonas gingivalis* exhibited higher relative abundance in oral biofilm of females (Benn et al., 2022). M. Minty, et al. found Porphyromonas and Capnocytophaga were overrepresented in the male salivary samples compared with female saliva (Minty et al., 2021). Our findings revealed significant differences in the oral microbiota of young women at different stages of periodontitis. In our study, the increased phylum Bacteroidetes, its order, family and genus Bacteroidales, *Prevotellaceae* and *Prevotella* in the young women with stage III periodontitis accounted for the periodontal destruction. *Prevotella* is a genus of Gram-negative anaerobic bacteria which have black colonies (Shah and Collins, 1990). The oral cavity has the greatest diversity of known species of *Prevotella* among body sites (Tett et al., 2021). *P. intermedia* is closely related to periodontal disease (Braga et al., 2010). In young women, due to fluctuations in SHH levels, the biological behavior of *P. intermedia* is also affected (Fteita et al., 2014; Cornejo Ulloa et al., 2021). Estradiol enhances the toxicity of *P. intermedia*, which is related to one of its virulence factors: protease dipeptidyl peptidase IV (Fteita et al., 2015). Murata and colleagues found that *P. intermedia* reduced the ALP activity in pre-osteoblasts (Murata et al., 1997). *P. intermedia* LPS stimulates the differentiation and activity of osteoclasts, and increases the release of matrix metalloproteinases to participate in bone destruction (Guan et al., 2009). However, the pathogenic mechanism of *P. intermedia* in periodontitis needs to be further explored.

The KEGG database revealed that protein processing in the ER was one of the most enriched pathways in young women with stage-III periodontitis. ER stress occurs if the intracellular demand for protein synthesis exceeds the capacity of the ER (Oakes and Papa, 2015). ER stress is closely related to bone metabolism (Chen et al., 2020; Xu et al., 2020). SAL is an inhibitor of eIF2α dephosphorylation, and reduces ER stress (Boyce et al., 2005). Previously, we found that ER stress occurred in alveolar bone formation after tooth extraction, and that SAL application promoted the expression and arrangement of type-I collagen and promoted osteogenic differentiation by reducing ER stress (Chen et al., 2020). Liu et al. also found that SAL regulated ER stress to promote angiogenesis and bone healing in ischemic osteonecrosis (Liu et al., 2017). SAL significantly alleviated the symptoms of postmenopausal osteoporosis, such as reduced bone mineral density and bone volume fraction (Li et al., 2019). Increased ER stress has also been observed in periodontitis (Domon et al., 2009). Therefore, we applied SAL to regulate ER stress in periodontitis and investigated if it promoted bone formation and reduced bone resorption in periodontitis *in vitro* and vivo.

Periodontitis was the inflammation and destruction of the tooth-supporting structures. The mechanisms begun with the release of endotoxins by bacteria, such as LPS (Kim et al., 2020), and included secretion of inflammatory mediators, activation of osteoclastogenesis, inhibition of osteogenesis, alveolar bone resorption by osteoclasts, and degradation of ligament fibres by matrix metalloproteinases (Kinane et al., 2017; Alqranei and Chellaiah, 2020; Zhou et al., 2021). Interesting, a single-cell RNA sequencing found the fractions of BMSCs, pre-osteoblasts, and osteoblasts were higher in periodontitis than after initial periodontal therapy (Chen et al., 2022). And in the periodontal microenvironment, bone-marrow mesenchymal cells are pluripotent stem cells, which can transform into pre-osteoblasts, osteoblasts and osteocytes. Therefore, we chose BMSCs as the object of further study on the mechanisms *in vitro*. First, we used different concentrations of SAL to explore the effect of its regulation of ER stress on osteogenesis: moderate ER stress promoted osteogenesis and upregulated expression of COL1A1, OCN and RUNX2. Studies have shown that ER stress exists in the pathogenesis of periodontitis (Domon et al., 2009). And relatively low concentration of SAL can slightly inhibit ER stress in periodontitis and promote osteogenesis, which was consistent with our previous research results (Chen et al., 2020). Contrary to our results, Yokota et al. discovered that SAL (1–5 μM) inhibited osteoclastogenesis and stimulated osteoblastogenesis in BMSCs (Yokota et al., 2013). Some scholars have also found that higher concentrations of SAL alleviated degradation of the extracellular matrix and the inflammatory response in human periodontal ligament cells induced by P. gingivalis LPS (Bai et al., 2016). This finding may be due to the inconsistent sensitivity of different cells to SAL and the complexity of inflammatory microenvironment. We applied SAL to a periodontitis model and observed its effect on bone resorption: alveolar bone resorption was inhibited and ALP activity was higher in the SAL group. Previously, we applied this concentration (0.143mg/kg) of SAL to a tooth-extraction model and found that the osteogenesis was improved in the extraction socket (Chen et al., 2020). Kimura et al. observed attenuation of alveolar bone resorption and loss of attachment level after 12 weeks of applying SAL (Kimura et al., 2021).

There is moderate evidence that *P. intermedia* is a pathogen associated with periodontitis (Gambin et al., 2021), but we found that its pathogenic component, LPS, promoted osteogenesis and upregulated expression of COL1A1, OCN and RUNX2 *in vitro*. Other scholars have also found that various components of bacteria have roles in promoting osteogenesis. Injection of killed bacterial species into the intramedullary canal of the tibia has been shown to increase bone mass significantly, and a low dose of killed bacteria has a stronger osteogenic effect (Croes et al., 2019). Lipoteichoic acid (cell-wall component of Gram-positive bacteria) can accelerate fracture healing in mice with bone defects (Hu et al., 2020). Scholars have also found that LPS of various types of bacteria can inhibit bone formation. LPS from *P. intermedia* and *P. gingivalis* has been shown to increase osteoclast differentiation and inhibit osteoblastic differentiation (Murata et al., 1997; Xing et al., 2010). Monasterio et al. found that Aggregatibacter actinomycetemcomitans LPS induced alveolar bone resorption and expression of receptor activator of nuclear factor kappa-B ligand in periodontitis (Monasterio et al., 2018). We found that *P. intermedia* LPS led to resorption of alveolar bone *in vivo*, which was inconsistent with the results of *in vitro* experiments. This finding may be related to a variation of cell types and a complex microenvironment in the periodontitis model.

Intervention of *P. intermedia* LPS promoted COL1A1 expression. Previously, we found that type-I collagen plays an important part in osteogenesis regulation through the Irs-1/miRNA-342 axis (Guo et al., 2016). Previously, we observed, using transmission electron microscopy, that if ER stress was inhibited, the arrangement of type-1 collagen around the ER was more ordered, which promoted bone formation (Chen et al., 2020). Therefore, changes in levels of type-I collagen are the key to the promotion of bone formation by mild inflammation, but the specific mechanism has yet to be investigated. The other cells contained in mechanisms of periodontitis need to further verify and study.

## Conclusions

The composition of oral microbiota varied in the different stages of periodontitis. Bacteria of the genus *Prevotella* could be biomarkers in young women with stage-III periodontitis, and their abundance was related to protein processing in an ER pathway.

## Data availability statement

The data presented in the study are deposited in NCBI under the BioProject accession number PRJNA893254.

## Ethics statement

The studies involving human participants were reviewed and approved by The Medical Ethics Committee, The Second Xiangya Hospital, Central South University, China. The patients/participants provided their written informed consent to participate in this study. The animal study was reviewed and approved by The Institutional Animal Care and Use Committee (IACUC), The Second Xiangya Hospital, Central South University, China.

## Author contributions

YZ and YunF performed experiments, designed and supervised experiments and analyzed data. YZ and QY performed experiments, collected data and co-wrote the manuscript. YG provided conceptual input and edited the manuscript. JH, YaoF, and ZO contributed to formal analysis and resources. JZ, YZ, and LT contributed to software and visualization. NC, MD, and XS performed animal experiments. All authors took part in drafting, revising or critically reviewing the article and gave final approval of the version to be published.

## Funding

This study was supported by the National Natural Science Foundation of China (81800788 and 81773339), Hunan Provincial Science and Technology Department (2017WK2041 and 2018SK52511), Health Commission of Hunan Province (202208043514), Natural Science Foundation of Hunan Province (2022JJ30062), Natural Science Foundation of Changsha City (kq2202403 and kq2202412), Fund for the Xiangya Clinical Medicine Database of Central South University (2014-ZDYZ-1-16), Education and Teaching Reform Research Project of Central South University (2020jy165-3), Research Project on Postgraduate Education and Teaching Reform of Central South University(2021JGB072), Open Sharing Fund for the Large-scale Instruments and Equipment of Central South University and the Fundamental Research Funds for the Central Universities of Central South University.

## Acknowledgments

The authors are grateful to the informants for taking the time to participate in this study.

## Conflict of interest

The authors declare that the research was conducted in the absence of any commercial or financial relationships that could be construed as a potential conflict of interest.

## Publisher’s note

All claims expressed in this article are solely those of the authors and do not necessarily represent those of their affiliated organizations, or those of the publisher, the editors and the reviewers. Any product that may be evaluated in this article, or claim that may be made by its manufacturer, is not guaranteed or endorsed by the publisher.
